# MRI-based spatio-temporal atlas of ganglionic eminence

**DOI:** 10.1186/s41747-026-00702-5

**Published:** 2026-04-14

**Authors:** Tommaso Ciceri, Andrea Righini, Letizia Squarcina, Adele Ferro, Cecilia Pini, Alessandra Marano, Valentina Tombola, Chiara Scacchetti, Nicola Persico, Simona Boito, Irene Cetin, Filippo Arrigoni, Cecilia Parazzini, Alessandra Bertoldo, Giorgio Conte, Fabio Maria Triulzi, Paolo Brambilla, Denis Peruzzo

**Affiliations:** 1 NeuroImaging Lab, Scientific Institute IRCCS Eugenio Medea, Bosisio Parini, Italy; 2 Department of Information Engineering, University of Padua, Padua, Italy; 3 Department of Radiology and Neuroradiology, Children’s Hospital V. Buzzi, Milan, Italy; 4 Department of Pathophysiology and Transplantation, University of Milan, Milan, Italy; 5 Department of Neurosciences and Mental Health, Fondazione IRCCS Ca’ Granda Ospedale Maggiore Policlinico, Milan, Italy; 6 Department of Woman, Child and Newborn, Fondazione IRCCS Ca’ Granda Ospedale Maggiore Policlinico, Milan, Italy; 7 Department of Clinical and Community Sciences, University of Milan, Milan, Italy; 8 Neuroscience Center, University of Padua, Padua, Italy; 9 Department of Services and Preventive Medicine, Fondazione IRCCS Ca’ Granda Ospedale Maggiore Policlinico, Milan, Italy

**Keywords:** Brain, Fetus, Ganglionic eminence, Gray matter, Magnetic resonance imaging

## Abstract

**Objective:**

Fetal brain magnetic resonance imaging (MRI) provides insights into the architecture of the human brain. Recently, an increasing interest has been posed on transient brain structures, such as the ganglionic eminence (GE), to better understand potential derailments or anomalies in neurodevelopment. In this work, we define a spatio-temporal atlas of the GE from 19 to 36 gestational weeks (GW) in a 0.5-mm isotropic resolution.

**Materials and methods:**

We extended the T2-weighted developing Human Connectome Project atlas with 19 and 20 GW and generated GE label maps spanning 19–36 GW. The GE label maps were generated via an averaging ensemble strategy of the segmentations performed by three expert neuroradiologists.

**Results:**

The segmentations conducted by the experts achieved 0.91 ± 0.06 Dice similarity coefficient throughout the whole range of GW, indicating a strong agreement in this task. The GE reached its maximum volume expansion at around 21 GW, followed by a pronounced reduction throughout pregnancy (*R*^2^ = 0.98, ranged 40‒500 mm^3^), highlighting an inverse relationship to the whole brain volume and cortical gray matter. This is accompanied by an increased number of small and fragmented components, correlating with known dynamics of GE migration toward target structures.

**Conclusion:**

The proposed spatio-temporal GE MRI atlas supports the monitoring during pregnancy of this fascinating brain structure. It may aid in better understanding prodromic signs of potential future clinical conditions attributable to GE alterations. Moreover, it could be used as a repository of knowledge to develop innovative atlas-based deep learning models for biometric, volumetric, and shape analysis.

**Relevance statement:**

The spatio-temporal fetal MRI atlas of the GE allows researchers to study its evolution and potential future clinical conditions attributable to GE alterations in pregnancy. The GE reached its maximum volume expansion around 21 GW, followed by a pronounced reduction throughout the pregnancy.

**Key Points:**

- The development of GE is a resource for monitoring pregnancy.
- We propose a spatio-temporal GE MRI atlas from 19 to 36 weeks of gestation.
- The GE reached its maximum expansion at around 21 weeks of gestation, followed by a progressive decline throughout pregnancy.

**Graphical Abstract:**

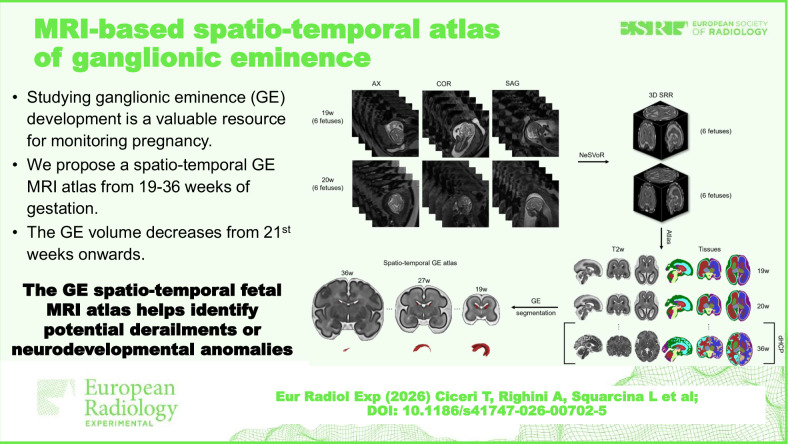

## Background

During pregnancy, the fetal brain undergoes significant development of its multiple brain structures, characterized by cellular proliferation, migration, and network formation [1]. An increasingly interesting transient structure of the fetal brain is the ganglionic eminence (GE).

**Fig. 1:**
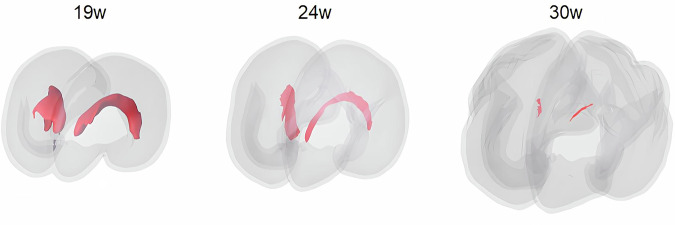
Render of the GE (in red), surrounded by the cortical gray matter (in light gray). The GEs in the angled anteroposterior view for the 19th, 24th, and 30th week of gestation

GE develops in the ventral telencephalon from the 5th week post-conception and disappears within the 35th gestational week (GW) [2]. It borders the lateral ventricles with a “C-shaped” distribution (Fig. 1) and can be subdivided into three main areas: lateral, medial, and caudal. These areas give rise to progenitor cells that later differentiate into a wide spectrum of interneurons that populate broad regions of the telencephalon, including the amygdala, basal ganglia, cerebral cortex, and olfactory bulb [3–5].

Anomalies within the GE may result in failure or insufficient cell proliferation and/or migration, leading to profound clinical consequences during postnatal life [6]. A summary of GE anomalies and their underlying causative processes was proposed in 2016 by Righini et al [7], along with other cortical, cerebellar, and white matter malformations. Furthermore, GE is also the most common site for neonatal intracerebral hemorrhage, particularly following preterm delivery [8]. However, to date, only remarkable and dramatic anomalies of GE shape and size have been reported [3], given the difficulty in detecting possible smaller volumetric abnormal variations.

Magnetic resonance imaging (MRI) represents a robust approach for investigating brain development and transient structures, such as GE, during gestation, as it provides noninvasive, high-resolution visualization of the fetal brain [9, 10]. However, its application is typically limited to examinations performed after 18 GWs, since earlier scans generally yield little additional information beyond that obtainable with transvaginal ultrasound, thereby reducing diagnostic utility at this stage [11]. In this scenario, several brain atlases [12–14] have been introduced to analyze the acquired data, facilitating inter-subject comparison and visualization. Brain atlases refer to a collection of data structured in a reference space that captures the anatomy (*e.g*., structural anatomy) or physiology (*e.g*., structural and functional connectivity) of a population. Fetal atlases are usually defined in the temporal domain (*i.e*., GWs), showing how specific data change and evolve over time. The term atlas is used to refer to two distinct images: the atlas reference image from brain MRI and the atlas label image, which denotes anatomical structures or tissues at each brain voxel [10, 15]. Labels can be particularly relevant for our understanding of fetal neurodevelopment as they provide insights into the brain architecture, and enable quantitative analysis of structural volumes and shapes.

In this work, we introduce a spatio-temporal MRI atlas of the GE from 19 to 36 GWs in a 0.5-mm isotropic resolution. To do so, we extended up to 19 GW a novel spatio-temporal MRI atlas from the developing Human Connectome Project (dHCP) [14], which is defined from 21 to 36 GWs. This novel atlas of dHCP consists of MRI structural (T2-weighted [T2w], T1-weighted) and diffusion-weighted (fractional anisotropy, mean diffusivity, radial diffusivity, direction-averaged diffusion-weighted image at *b*-value of 1,000 s/mm^2^, fiber orientation distribution) channels, tissue segmentation, affine transformations, and label description. Here, we focused only on the T2w MRI channel, adding to the tissue segmentation maps the GE label. The GE atlas label images, along with the relative atlas reference T2w images, are made publicly available (https://github.com/tommaso-ciceri/MRI-based-spatio-temporal-atlas-of-ganglionic-eminence). This atlas provides a valuable resource to study the GE development through gestations and ease localization of its anomalies, which nevertheless may still contribute to major neurodevelopmental impairment.

## Materials and methods

### Data

The fetal brain dataset used for the MRI atlas construction was collected at the Scientific Institute IRCCS Fondazione Ca’ Granda Ospedale Maggiore Policlinico (Milan, Italy). Mothers were recruited as part of a broader research project that included a healthy cohort, and, therefore, underwent MRI examinations without any clinical indication. Exclusion criteria for mothers include: (1) twin pregnancy; (2) history of perinatal adverse events; (3) infective or autoimmune diseases; (4) use of systemic corticosteroids; and (5) congenital, genetic, or neurological disorders. Exclusion criteria for the fetus include congenital and genetic disorders, and the presence of brain malformation at MRI. The procedures were approved by the institutional ethical review boards of the hospital, and all women signed an informed consent for the research use of their data.

Examinations were performed using a phased-array abdominal coil with a 3-T Achieva d-Stream scanner (Philips Healthcare, Best, The Netherlands) within 45 min, and neither contrast medium nor sedation was applied. Fetal brain imaging data included T2w turbo spin-echo sequences of the fetal head in three orthogonal planes (slice thickness 2.5 mm; repetition time 3500 ms; echo time 180 ms; field of view, 160 × 160 mm; in-plane resolution, 0.44 × 0.44 mm).

The processing pipeline consisted of slice-wise motion-correction and super-resolution reconstruction at 0.5-mm isotropic resolution using NeSVoR [16]. Subsequently, the derived fetal brain reconstructions were brain-masked by binarizing and manually refining the tissue segmentation masks generated using the FetalSynthSeg tool [17]. The total selected cohort includes 6 fetuses at 19 weeks (from 19 + 0 to 19 + 6) and 6 at 20 weeks (20 + 0 to 20 + 5), all characterized by excellent image quality (Supplementary Material Fig. S1).

### Atlases construction

The fetal brain spatio-temporal atlas of dHCP [14] (10.12751/g-node.ysgsy1) was constructed using the MIRTK toolbox with multichannel (T2w + cortex) guided registration, similar to the neonatal atlas [18]. The fetal brain was segmented into 19 regions of interest (ROIs), with separation of right and left structures, following fetal brain histology atlases [19, 20]. The ROIs were drawn by manually refining the segmentations obtained with the dHCP Draw-EM neonatal pipeline [21] combined with registration across timepoints to preserve structural coherence throughout gestation.

In this study, we used the open-source Advanced Normalization Tools software (ANTs, https://stnava.github.io/ANTs//) [22] to extend the fetal brain atlas of dHCP with the 19th and 20th GW templates. Notably, we generated the atlas reference T2w image for 19 and 20 GWs using an iterative approach (antsMultivariateTemplateConstruction2.sh), including all 6 fetuses available for each GW. This approach enabled a multichannel (T2w + cortex) registration using a nonlinear transformation (*i.e*., symmetric normalization), with the target image for each GW set to the T2w atlas of the subsequent week. For example, the 20-week atlas was used as the target for constructing the 19-week atlas. Then, we performed a guided rigid registration of the generated 19- and 20-week T2w atlas images to the 21-week T2w image of the dHCP fetal atlas [14] to account for any misalignment error. The corresponding atlas label images for 19 and 20 GW, including the 19 ROIs, were derived by applying the calculated transformations. The 19 ROIs comprised the intracranial volume (ICV) and bilateral structures, including the cerebellum, basal ganglia and thalami, brainstem, cortical gray matter (cGM), white matter, lateral ventricles, third and fourth ventricles, cavum septum pellucidum, and external cerebrospinal fluid.

### Annotation protocol of GE

The GE was segmented on the T2w atlas reference image at each GW investigated, specifically on the constructed T2w atlas image for 19–20 GWs and on the dHCP T2w atlas image for 21–36 GWs [14]. The volumetric segmentation followed a three-step procedure: coarse segmentation, manual refinement, and label fusion.

In the first step, an initial coarse segmentation of the GE was obtained through the automatic region-growing approach of ITK-SNAP [23]. Prior to its application, fetal brain image smoothing was applied using a lower band-pass filter (< 500), effectively removing all intensities above 500 to enhance boundary definition and reduce noise. Multiple seed points were strategically placed within the GE to ensure accurate region expansion while maintaining anatomical precision. The segmentation process was executed over 1,000 iterations, allowing the region to grow progressively while adapting to intensity variations and structural boundaries.

In the second step, the volume of the GE of both hemispheres was manually refined voxel by voxel as displayed by all three-planar slices by three senior pediatric neuroradiologists (F.A., C.P., and A.R.) with more than ten years of experience, who performed the task independently using ITK-SNAP [23]. A preliminary session was held to discuss the definition of the GE border by scrutinizing anatomical atlases of the fetal brain. In particular, the consensus aimed at pinpointing the more challenging areas to assess, namely the posterior-caudal “tail” of the GE and its anterior-frontal tip. During the second and third trimesters, the general GE shape could grossly resemble a “slice-wedge of a tangerine” with its anterior half being more globular and larger than the posterior one. Therefore, an anterior “nose” and a posterior “tail” could be identifiable in the “tangerine slice-wedge”. In detail, it was decided to interrupt the tracing of the GE caudal and head border at the point where the tip of the hypointensity continued as a thin ribbon of similar signal but with parallel margins (that is, the putative site where the “generic” germinal-matrix periventricular remnant is supposed to begin).

In the third step, an ensemble averaging approach was adopted to fuse the three different atlas label maps of each operator, obtaining a probability map of the GE. Therefore, a probability threshold of 0.5 was applied to derive the final binary GE label for each GW.

### Atlas assessment

The spatio-temporal GE MRI atlas was evaluated through multiple complementary analyses.

First, we assessed the consistency of the GE segmentations performed independently by the three neuroradiologists across 19–36 GWs. Inter-rater agreement was quantified using three metrics: the Dice similarity coefficient (DSC) to measure the spatial overlap [13, 24], the volume similarity (VS) to measure the volume difference [24], and the Hausdorff distance (HD) to quantify the contour distance. All metrics were calculated using the EvaluateSegmentation tool [25].

Second, we evaluated the accuracy of the 19- and 20-week atlas GE labels by comparing them with the manually performed GE segmentations of the original fetuses (6 per GW, used for atlas construction). Each fetus was registered to the corresponding atlas image using the symmetric normalization registration algorithm (affine + deformable transformation) [22], and similarity between atlas- and subject-level GE segmentations was assessed with DSC, VS, and HD [25].

Third, we qualitatively and quantitatively analyzed GE evolution across GWs. Qualitatively, morphological patterns of the GE were visually inspected by neuroradiologists, focusing on spatial distribution and structural continuity. Quantitatively, the total GE volume was measured, and its growth trajectory compared to ICV and cGM volume (primary GE migration target). Local volumetric changes were further captured using the logarithmic Jacobian determinant of symmetric normalization-derived deformation fields between consecutive atlas T2w images [22]. Positive log-Jacobian values indicate local expansion, while negative values reflect contraction.

Finally, we assessed the 19 ROIs across 19–36 GWs to evaluate the volume consistency of the constructed 19- and 20-week atlas with respect to the dHCP fetal atlas [14].

## Results

**Fig. 2:**
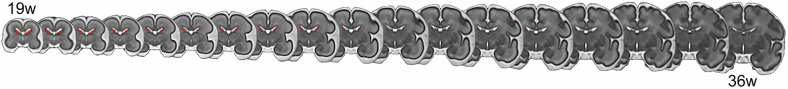
T2w images of the fetal brain with the GE labels (in red) covering GWs from 19 to 36

The atlas GE label maps, together with the T2w reference images covering 19–36 GWs, are shown in Fig. 2.

Inter-rater consistency of GE segmentations, performed by the three neuroradiologists, showed strong overall agreement across GWs. Notably, we achieved a global average DSC of 0.91 ± 0.06 (mean ± standard deviation); VS of 0.75 ± 0.11; and HD of 11.05 ± 3.89 (Table 1). A slight reduction in agreement was observed from the beginning of the third trimester onwards, reflecting the increased difficulty of delineating the GE as it migrates toward the target structures (*e.g*., cortex) and its contrast with surrounding tissue decreases.

**Table 1:** Inter-rater consistency of GE segmentations manually performed by the three neuroradiologists in the 19‒36 GWs

Gestational age [weeks]	DSC ↑ [mean ± SD]	VS ↑ [mean ± SD]	HD ↓ [mean ± SD]
19	0.85 ± 0.08	0.97 ± 0.03	3.05 ± 0.09
20	0.95 ± 0.01	0.92 ± 0.04	4.94 ± 0.61
21	0.95 ± 0.03	0.90 ± 0.06	4.33 ± 0.63
22	0.94 ± 0.04	0.89 ± 0.07	8.61 ± 3.27
23	0.92 ± 0.05	0.84 ± 0.09	9.72 ± 4.49
24	0.97 ± 0.01	0.96 ± 0.03	8.94 ± 5.63
25	0.97 ± 0.02	0.95 ± 0.03	9.01 ± 4.14
26	0.99 ± 0.01	0.98 ± 0.02	3.24 ± 0.87
27	0.96 ± 0.02	0.89 ± 0.04	4.96 ± 2.62
28	0.94 ± 0.03	0.88 ± 0.08	6.73 ± 2.29
29	0.91 ± 0.06	0.89 ± 0.08	18.61 ± 4.96
30	0.87 ± 0.11	0.74 ± 0.23	14.65 ± 4.43
31	0.82 ± 0.12	0.65 ± 0.24	16.21 ± 4.36
32	0.86 ± 0.10	0.73 ± 0.20	11.14 ± 4.61
33	0.92 ± 0.07	0.83 ± 0.14	12.60 ± 6.06
34	0.86 ± 0.09	0.72 ± 0.18	18.31 ± 6.21
35	0.87 ± 0.09	0.73 ± 0.19	21.12 ± 8.32
36	0.87 ± 0.09	0.75 ± 0.17	22.67 ± 6.39

*DSC* Dice similarity coefficient, *HD* Hausdorff distance, *SD* Standard deviation, *VS* Volume similarity, ↑ Higher is better, ↓ Lower is better

The atlas GE segmentations at 19 and 20 GWs showed good agreement with the manual subject-level annotations, confirming accurate atlas construction (Table 2). Additionally, voxel intensity distributions inside and outside the GE in the atlas space at these GWs are reported in the Supplementary Material (Fig. S2), showing a uniform distribution within the GE with respect to the surrounding tissues.

**Table 2:** Similarity between atlas- and manual subject-level GE segmentations at 19 and 20 GWs

Gestational age [weeks]	DSC ↑ [mean ± SD]	VS ↑ [mean ± SD]	HD ↓ [mean ± SD]
19	0.70 ± 0.03	0.94 ± 0.04	11.5 ± 0.8
20	0.78 ± 0.06	0.89 ± 0.1	8.1 ± 3.2

*DSC* Dice similarity coefficient, *HD* Hausdorff distance, *SD* Standard deviation, *VS* Volume similarity, ↑ Higher is better, ↓ Lower is better

**Fig. 3:**
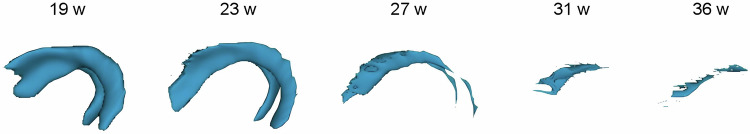
Morphology of the GEs’ evolution during pregnancy. The three-dimensional reconstructions are displayed in the left lateral view at GWs 19, 23, 27, 31, and 36

Qualitative analysis of atlas GE segmentations (Fig. 3) shows the characteristic “C-shape” morphology bordering the lateral ventricles before 24 GW. A progressive thinning of the caudal portion emerges around 26–27 GW, followed by reductions in the medial and lateral regions, which become apparent after 31 GW, consistent with the known spatio-temporal dynamics of GE migration.

**Fig. 4:**
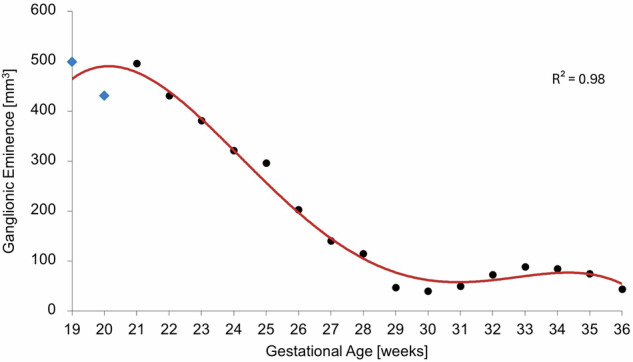
Scatterplot of GE volume across 19-to-36 GWs, with a polynomial fit capturing the overall trend

Quantitative analysis revealed that GE volume reaches its maximum expansion around 21 GW, followed by a pronounced decrease throughout the late second trimester before stabilizing during the third trimester. This trajectory was best captured by a polynomial fit (4th-degree; *R*^2^ of 0.98), with GE volumes ranging from 40 and 500 mm^3^ (Fig. 4). No hemispheric GE differences were observed (*r* = 0.99).

**Fig. 5:**
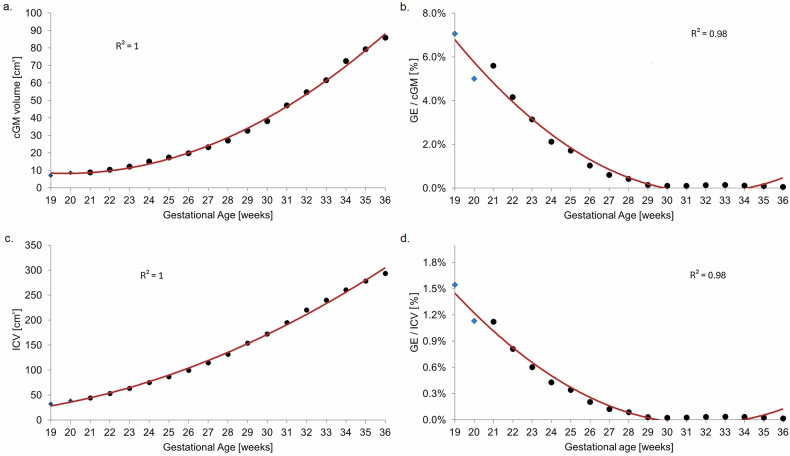
Scatterplots showing (**a**) cGM, and (**c**) ICV, together with their respective ratios to the GE volume (**b**, **d**), across 19–36 GWs, with polynomial fits capturing the overall trends

The relationship between GE and one of its migration targets, the cGM, followed an exponential trajectory. Specifically, cGM volume increased exponentially throughout gestation (*R*² = 1.00, Fig. 5a), while GE volume exhibited a sharp reduction relative to cGM during the second trimester, followed by a slower decline in the third trimester (*R*² = 0.98, Fig. 5b). A similar exponential trend was observed between GE and ICV (*R*² = 0.98, Fig. 5d), as also ICV volume increased exponentially throughout gestation (*R*² = 1.00, Fig. 5c). These opposing dynamics were most evident between 21 and 23 GWs, when GE volume decreased markedly as cGM and ICV expanded rapidly.

**Fig. 6:**
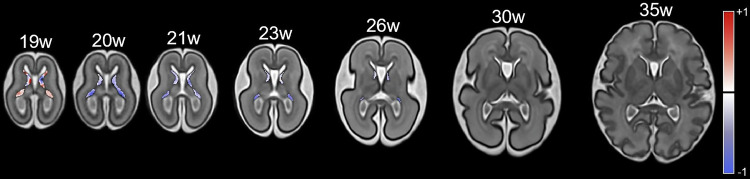
Log-Jacobian maps within the GE across GWs, overlaid on axial T2w templates. Positive values, indicating local expansion, are shown as shades of red (from white to dark red), while negative values, indicating local shrinkage, are shown as shades of blue (from white to dark blue)

Log-Jacobian analysis revealed an initial phase of local expansion within the GE during early gestation, followed by a progressive shift toward contraction as gestation advanced (Fig. 6). Regression was most pronounced in the caudal and medial (outer) regions, consistent with the expected trajectory of GE maturation.

**Fig. 7:**
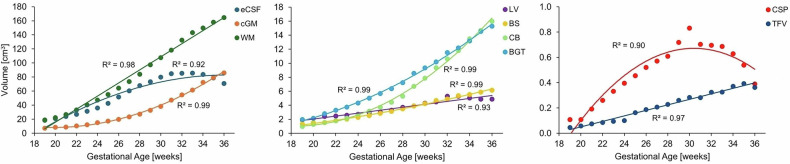
Scatterplot of volumetric growth trajectories for white matter (WM), lateral ventricles (LV), cerebellum (CB), 3rd and 4th ventricles (TFV), brainstem (BS), basal ganglia and thalami (BGT), cavum septum pellucidum (CSP), and external cerebrospinal fluid (eCSF) across GWs

The volumetric analysis of the ROIs across 19-36 GWs revealed distinct developmental trajectories (Fig. 7). Structures such as the cerebellum, basal ganglia and thalami, brainstem, and cGM exhibited exponential growth patterns, whereas white matter, lateral ventricles, and third and fourth ventricles increased linearly with gestational age. The cavum septum pellucidum and external cerebrospinal fluid displayed parabolic trajectories, with volumes reaching maximum expansion around 30 GW and decreasing thereafter. The high goodness-of-fit of these trajectory models (*R*^2^ > 0.9) confirms that the 19- and 20-week atlases are consistent with the growth patterns established by the dHCP fetal atlas, supporting their integration into the spatio-temporal framework.

**Fig. 8:**
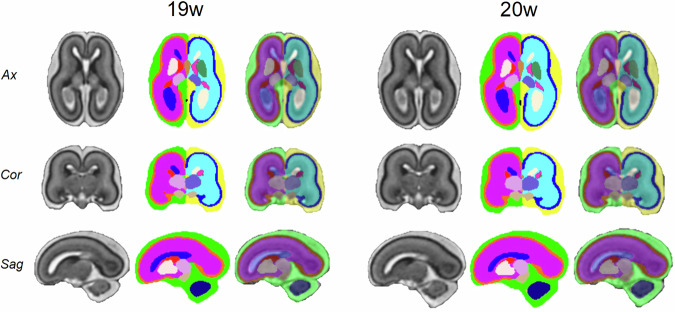
Fetal brain atlases at 19 and 20 GWs. Structural T2w images, tissue label maps, and their overlays are displayed in the three orthogonal views (axial, coronal, and sagittal). Tissue maps include GE labels (red-colored for the right hemisphere and pink-colored for the left hemisphere)

The corresponding tissue segmentation maps, including the GE label, are shown in Fig. 8 alongside their T2w images. For comparison, the native tissue maps (without GE) are provided in the Supplementary Material (Fig. S3).

## Discussion

In this study, we extended to the 19th GW the fetal brain spatio-temporal atlas of dHCP, incorporating an atlas label map of the GE for each GW. The second trimester, particularly around 18–24 weeks of gestation, is a crucial period for fetal MRI analysis because the central nervous system has developed enough for detailed imaging, and from a clinical standpoint, it provides critical information for both parental decision-making and perinatal management [11, 26].

The GE is a transient brain structure that undergoes a substantial change in size and shape during pregnancy. Notably, its volume decreases during GWs due to ongoing neuronal migration towards target brain structures (*e.g*., the cortex). This is accompanied by an increased number of small and fragmented components, which present significant challenges for neuroradiologists attempting to segment it manually at later GWs. Furthermore, there is a visible thinning of the caudal GE prior to the medial and lateral portions (Fig. 3). However, the manual segmentations performed by the three neuroradiologists across GWs showed a very high level of consistency, indicating strong spatial agreement and reproducibility of the task.

We show that the GE volume reaches its maximum expansion around the 21st week of gestation, consistent with a recent T2w *in vivo* study [6] and a diffusion tensor imaging postmortem study [27]. Our findings further indicate a marked decline in GE volume during the late second trimester, followed by relative stabilization throughout the third trimester, aligning with physiological neurodevelopmental expectations. A direct comparison with the *in vivo* fetal data reported in 2023 by Stuempflen et al [6] further supports this interpretation: absolute GE volumes in our atlas fall within the ranges they report during early gestation and follow a similarly decreasing trajectory thereafter, albeit with a steeper decline in late gestation. Likewise, the GE-to-ICV ratio shows comparable developmental patterns. In contrast, some previous *in vivo* volumetric studies [28, 29] report a continued increase in GE volume across gestation, raising doubts about the true developmental trajectory of this transient structure. The discrepancy may stem from differences in GE labeling methodology: whereas both Stuempflen et al [6] and our study employed expert manual GE segmentations, Machado-Rivas et al [29] used atlas-based label propagation, and Sousa et al [28] trained a deep learning model on labels also obtained via atlas propagation. Such atlas-based approaches may be less suited to accurately capturing the geometry of a transient structure like the GE, whose shape and boundaries change markedly across gestations.

The proposed spatio-temporal atlas at the 19- and 20-week gestation is constructed by adopting 6 subjects for each week of gestation. According to some previous studies [30–33], a time-weighted kernel regression algorithm [34], accounting for fetuses at the nearest gestational ages to increase the sample for each GW investigated, was adopted for its construction. On one hand, our week-based approach enables us to build a specific atlas reference image, preventing some implicit temporal consistency between brain structures and highlighting transitory structures peculiar only in that GW. On the other hand, this approach has the limitation that each atlas reference image may be based only on a few subjects, and images referring to consecutive GW periods may be quite different. However, combining images from different GWs, as performed in several studies [13, 35–40], must be carefully considered to prevent misleading structures definition and standardize the population variability between GWs, especially in pathological conditions. Furthermore, our approach enabled us to expand an existing atlas, despite the unavailability of its raw data.

The fetal brain atlas still plays a crucial role as a repository of anatomical priors that could be used for the generation of training datasets with labels propagated from the atlas. According to a recent method [41], these datasets can be employed to train innovative atlas-based deep learning models for large-scale biometric, volumetric, and shape analysis. In terms of the limitations, our current extended atlas label image includes only 21 ROIs for each GW. Previously introduced atlases (*e.g*., CRL-atlas [13]) identified tens of regions for each major brain structure, including later GWs (*e.g*., 37th and 38th). However, some concerns related to the reliability and reproducibility of these segmentations may arise from the fact that the fetal brain size ranges from 103 cm^3^ at 22–24 weeks to 319 cm^3^ at 32–34 weeks [42], and the slice-to-volume reconstructed images are obtained from raw data characterized by a voxel size ranging between 0.4 and 1.0 mm, and with a large slice thickness ranging between 2 and 4 mm [15, 43]. Surely, further subdivision of brain anatomy into structures not included in the atlas label map (*e.g*., white matter in subplate and ventricular zone in the second trimester of pregnancy), as well as the differentiation of existing brain structures (*e.g*., the GE in the caudal, frontal, and medial regions), should be undertaken but maintaining a good trade-off between resolution, reliability, and reproducibility. In this regard, both the combination of distinct anatomical MRI acquisition sequences (*e.g*., diffusion images) and the quality of fetal brain reconstructions should be considered to better characterize brain structures and achieve more accurate segmentations. Since our atlas is based exclusively on T2w data acquired at a single center, sequence-dependent variations in tissue contrast and differences in acquisition protocols across scanners may affect its generalizability and influence atlas-derived measurements. Furthermore, normative curves of the GE developmental trajectory were not defined, as the sample size in the present study is limited. Establishing robust normative references would require accounting for population variables such as ethnicity and sex, as well as substantially larger cohorts to reliably estimate percentile ranges and confidence intervals.

In conclusion, the prenatal period is a critical window for individual brain development. Investigating the early developmental trajectories of the GE should be a priority for the scientific community to maximize our understanding of the developing brain and potentially related devastating pathologies. In this scenario, the proposed spatio-temporal GE atlas provides a valuable resource for both research and clinical assessment.

## Supplementary information

**Additional file 1**: **Figure S1**: Image quality assessment of the fetuses included in atlas construction at 19 and 20 GWs. Each fetal T2w reconstruction is shown in the three orthogonal views (axial, coronal, and sagittal). w = week, d = days. **Figure S2**: Normalized voxel intensity distributions of the GE probability maps at 19 and 20 gestational weeks. Solid lines represent voxels within the GE in the atlas space, while dashed lines correspond to voxels outside the GE mask in the atlas space. Probability values were normalized to the [0, 1] range. **Figure S3**: Atlas of fetal brains between 19 and 36 weeks of gestation. The structural images and the label maps are reported in the three orthogonal views (axial, coronal, and sagittal) for each week.

## Data Availability

The spatio-temporal GE MRI atlas is provided *via* GitHub (https://github.com/tommaso-ciceri/MRI-based-spatio-temporal-atlas-of-ganglionic-eminence).

## Abbreviations

ANTs: Advanced normalization tools
cGM: Cortical gray matter
dHCP: Developing human connectome project
DSC: Dice similarity coefficient
GE: Ganglionic eminence
GW: Gestational week(s)
HD: Hausdorff distance
ICV: Intracranial volume
MRI: Magnetic resonance imaging
ROI: Region of interest
T2w: T2-weighted
VS: Volume similarity

## References

[1] Serati M, Delvecchio G, Orsenigo G et al (2019) The role of the subplate in schizophrenia and autism: a systematic review. Neuroscience 408:58–67. 10.1016/j.neuroscience.2019.03.049 30930130 10.1016/j.neuroscience.2019.03.049

[2] Boitor-Borza D, Turcu F, Farcasanu S et al (2021) Early development of human ganglionic eminences assessed in vitro by using 7.04 Tesla micro-MRI—a pilot study. Med Pharm Rep 94:35–42. 10.15386/mpr-1715 33629046 10.15386/mpr-1715 PMC7880059

[3] Righini A, Frassoni C, Inverardi F et al (2013) Bilateral cavitations of ganglionic eminence: a fetal MR imaging sign of halted brain development. AJNR Am J Neuroradiol 34:1841–1845. 10.3174/ajnr.A3508 23598830 10.3174/ajnr.A3508 PMC7965643

[4] Nery S, Fishell G, Corbin JG (2002) The caudal ganglionic eminence is a source of distinct cortical and subcortical cell populations. Nat Neurosci 5:1279–1287. 10.1038/nn971 12411960 10.1038/nn971

[5] Kepecs A, Fishell G (2014) Interneuron cell types are fit to function. Nature 505:318–326. 10.1038/nature12983 24429630 10.1038/nature12983 PMC4349583

[6] Stuempflen M, Taymourtash A, Kienast P et al (2023) Ganglionic eminence: volumetric assessment of transient brain structure utilizing fetal magnetic resonance imaging. Ultrasound Obstet Gynecol 62:405–413. 10.1002/uog.26232 37099530 10.1002/uog.26232

[7] Righini A, Cesaretti C, Conte G et al (2016) Expanding the spectrum of human ganglionic eminence region anomalies on fetal magnetic resonance imaging. Neuroradiology 58:293–300. 10.1007/s00234-015-1622-5 26608601 10.1007/s00234-015-1622-5

[8] Tan AP, Svrckova P, Cowan F et al (2018) Intracranial hemorrhage in neonates: a review of etiologies, patterns and predicted clinical outcomes. Eur J Paediatr Neurol 22:690–717. 10.1016/j.ejpn.2018.04.008 29731328 10.1016/j.ejpn.2018.04.008

[9] Manganaro L, Capuani S, Gennarini M et al (2023) Fetal MRI: What’s new? A short review. Eur Radiol Exp 7:41. 10.1186/s41747-023-00358-5 37558926 10.1186/s41747-023-00358-5 PMC10412514

[10] Ciceri T, Casartelli L, Montano F et al (2024) Fetal brain MRI atlases and datasets: a review. Neuroimage 292:120603. 10.1016/j.neuroimage.2024.120603 38588833 10.1016/j.neuroimage.2024.120603 PMC12064217

[11] Prayer D, Malinger G, De Catte L et al (2023) ISUOG practice guidelines (updated): performance of fetal magnetic resonance imaging. Ultrasound Obstet Gynecol 61:278–287. 10.1002/uog.26129 36722431 10.1002/uog.26129 PMC10107509

[12] Bagheri M, Velasco-Annis C, Wang J et al (2025) An MRI atlas of the human fetal brain: reference and segmentation tools for fetal brain MRI analysis. Preprint at https://arxiv.org/abs/2508.15034 10.1038/s41597-026-07608-2 42386757

[13] Gholipour A, Rollins CK, Velasco-Annis C et al (2017) A normative spatiotemporal MRI atlas of the fetal brain for automatic segmentation and analysis of early brain growth. Sci Rep 7:476. 10.1038/s41598-017-00525-w 28352082 10.1038/s41598-017-00525-w PMC5428658

[14] Uus A, Kyriakopoulou V, Cordero Grande L et al (2023) Multi-channel spatio-temporal MRI atlas of the normal fetal brain development from the developing human connectome project. G-Node. 10.12751/g-node.ysgsy1/

[15] Makropoulos A, Counsell SJ, Rueckert D (2018) A review on automatic fetal and neonatal brain MRI segmentation. Neuroimage 170:231–248. 10.1016/j.neuroimage.2017.06.074 28666878 10.1016/j.neuroimage.2017.06.074

[16] Xu J, Moyer D, Gagoski B et al (2023) NeSVoR: implicit neural representation for slice-to-volume reconstruction in MRI. IEEE TMI 42:1707–1719. 10.1109/TMI.2023.3236216 10.1109/TMI.2023.3236216 PMC10287191 37018704

[17] Zalevskyi V, Sanchez T, Roulet M (2024) Improving cross-domain brain tissue segmentation in fetal MRI with synthetic data. Paper presented at the MICCAI 2024. Lecture notes in computer science, vol. 15001. MICCAI, pp 437–447

[18] Schuh A, Makropoulos A, Robinson EC et al (2018) Unbiased construction of a temporally consistent morphological atlas of neonatal brain development. Preprint at 10.1101/251512

[19] Bayer SA, Altman J (2003) The human brain during the third trimester. CRC Press, Boca Raton, p 392.

[20] Bayer SA, Altman J (2005) The human brain during the second trimester. CRC Press, Boca Raton, p 392.

[21] Makropoulos A, Robinson EC, Schuh A et al (2018) The developing human connectome project: a minimal processing pipeline for neonatal cortical surface reconstruction. Neuroimage 173:88–112. 10.1016/j.neuroimage.2018.01.054 10.1101/125526 PMC6783314 29409960

[22] Avants BB, Tustison NJ, Wu J et al (2011) An open source multivariate framework for n-tissue segmentation with evaluation on public data. Neuroinformatics 9:381–400. 10.1007/s12021-011-9109-y 10.1007/s12021-011-9109-y PMC3297199 21373993

[23] Yushkevich PA, Piven J, Hazlett HC et al (2006) User-guided 3D active contour segmentation of anatomical structures: significantly improved efficiency and reliability. Neuroimage 31:1116–1128. 10.1016/j.neuroimage.2006.01.015 10.1016/j.neuroimage.2006.01.015 16545965

[24] Dong C, Loy CC, Tang X (2016) Accelerating the super-resolution convolutional neural network. Paper presented at the computer vision—ECCV 2016. Lecture notes in computer science, vol. 9906. ECCV, pp 391–407

[25] Taha AA, Hanbury A (2015) Metrics for evaluating 3D medical image segmentation: analysis, selection, and tool. BMC Med Imaging 15:29. 10.1186/s12880-015-0068-x 10.1186/s12880-015-0068-x PMC4533825 26263899

[26] Moltoni G, Talenti G, Righini A (2021) Brain fetal neuroradiology: a beginner’s guide. Transl Pediatr 10:1065–1077. 10.21037/tp-20-293 10.21037/tp-20-293 PMC8107845 34012856

[27] Huang H, Xue R, Zhang J et al (2009) Anatomical characterization of human fetal brain development with diffusion tensor magnetic resonance imaging. J Neurosci 29:4263–4273. 10.1523/JNEUROSCI.2769-08.2009 19339620 10.1523/JNEUROSCI.2769-08.2009 PMC2721010

[28] Sousa HS, Fukami-Gartner A, Alena UU (2023) A deep learning approach for segmenting the subplate and proliferative zones in fetal brain MRI. In: Paper presented at the PIPPI 2023. Lecture notes in computer science, vol. 14246 (CoLab, pp 17–27)

[29] Machado-Rivas F, Gandhi J, Choi JJ et al (2022) Normal growth, sexual dimorphism, and lateral asymmetries at fetal brain MRI. Radiology 303:162–170. 10.1148/radiol.211222 34931857 10.1148/radiol.211222 PMC8962825

[30] Habas PA, Kim K, Corbett-Detig JM et al (2010) A spatiotemporal atlas of MR intensity, tissue probability and shape of the fetal brain with application to segmentation. Neuroimage 53:460–470. 10.1016/j.neuroimage.2010.06.054 20600970 10.1016/j.neuroimage.2010.06.054 PMC2930902

[31] Clouchoux C, Kudelski D, Gholipour A et al (2012) Quantitative *in vivo* MRI measurement of cortical development in the fetus. Brain Struct Funct 217:127–139. 10.1007/s00429-011-0325-x 21562906 10.1007/s00429-011-0325-x

[32] Xia J, Wang F, Benkarim OM et al (2019) Fetal cortical surface atlas parcellation based on growth patterns. Hum Brain Mapp 40:3881–3899. 10.1002/hbm.24637 31106942 10.1002/hbm.24637 PMC6865595

[33] Urru A, Nakaki A, Benkarim O et al (2023) An automatic pipeline for atlas-based fetal and neonatal brain segmentation and analysis. Comput Methods Programs Biomed 230:107334. 10.1016/j.cmpb.2023.107334 36682108 10.1016/j.cmpb.2023.107334

[34] Nadaraya EA (1964) On estimating regression. Theory of probability & its applications. Sci Res 9:141–142

[35] Serag A, Kyriakopoulou V, Rutherford MA et al (2012) A multi-channel 4D probabilistic atlas of the developing brain: application to fetuses and neonates. Annals of the BMVA 3:1–14

[36] Dittrich E, Riklin Raviv T, Kasprian G et al (2014) A spatio-temporal latent atlas for semi-supervised learning of fetal brain segmentations and morphological age estimation. Med Image Anal 18:9–21. 10.1016/j.media.2013.08.004 24080527 10.1016/j.media.2013.08.004

[37] Wright R, Makropoulos A, Kyriakopoulou V et al (2015) Construction of a fetal spatio-temporal cortical surface atlas from in utero MRI: application of spectral surface matching. Neuroimage 120:467–480. 10.1016/j.neuroimage.2015.05.087 26070259 10.1016/j.neuroimage.2015.05.087

[38] Khan S, Vasung L, Marami B et al (2019) Fetal brain growth portrayed by a spatiotemporal diffusion tensor MRI atlas computed from in utero images. Neuroimage 185:593–608. 10.1016/j.neuroimage.2018.08.030 30172006 10.1016/j.neuroimage.2018.08.030 PMC6289660

[39] Xu X, Sun C, Sun J et al (2022) Spatiotemporal atlas of the fetal brain depicts cortical developmental gradient. J Neurosci 42:9435–9449. 10.1523/JNEUROSCI.1285-22.2022 36323525 10.1523/JNEUROSCI.1285-22.2022 PMC9794379

[40] Chen R, Sun C, Liu T et al (2022) Deciphering the developmental order and microstructural patterns of early white matter pathways in a diffusion MRI based fetal brain atlas. Neuroimage 264:119700. 10.1016/j.neuroimage.2022.119700 36270621 10.1016/j.neuroimage.2022.119700

[41] Uus AU, Kyriakopoulou V, Makropoulos A et al (2023) BOUNTI: brain volumetry and automated parcellation for 3D fetal MRI. Preprint at 10.1101/2023.04.18.537347

[42] Tran CBN, Nedelec P, Weiss DA et al (2023) Development of gestational age-based fetal brain and intracranial volume reference norms using deep learning. AJNR Am J Neuroradiol 44:82–90. 10.3174/ajnr.A7747 36549845 10.3174/ajnr.A7747 PMC9835919

[43] Ciceri T, Squarcina L, Pigoni A et al (2023) Geometric reliability of super-resolution reconstructed images from clinical fetal MRI in the second trimester. Neuroinformatics 21:549–563. 10.1007/s12021-023-09635-5 37284977 10.1007/s12021-023-09635-5 PMC10406722

