# Supplementary material for: MRI-based spatio-temporal atlas of ganglionic eminence
Source: Eur Radiol Exp. 2026 Apr 14;10:47. doi: 10.1186/s41747-026-00702-5 (PMC13079254; doi:10.1186/s41747-026-00702-5)
Supplement: Supplementary file 1 — Additional file 1: Figure S1: Image quality assessment of the fetuses included in atlas construction at 19 and 20 GWs. Each fetal T2w reconstruction is shown in the three orthogonal views (axial, coronal, and sagittal). w = week, d = days. Figure S2: Normalized voxel intensity distributions of the GE probability maps at 19 and 20 gestational weeks. Solid lines represent voxels within the GE in the atlas space, while dashed lines correspond to voxels outside the GE mask in the atlas space. Probability values were normalized to the [0, 1] range. Figure S3: Atlas of fetal brains between 19 and 36 weeks of gestation. The structural images and the label maps are reported in the three orthogonal views (axial, coronal, and sagittal) for each week. [file 41747_2026_702_MOESM1_ESM.pdf]

MRI-based spatio-temporal atlas of ganglionic eminence

ELECTRONIC SUPPLEMENTARY MATERIAL

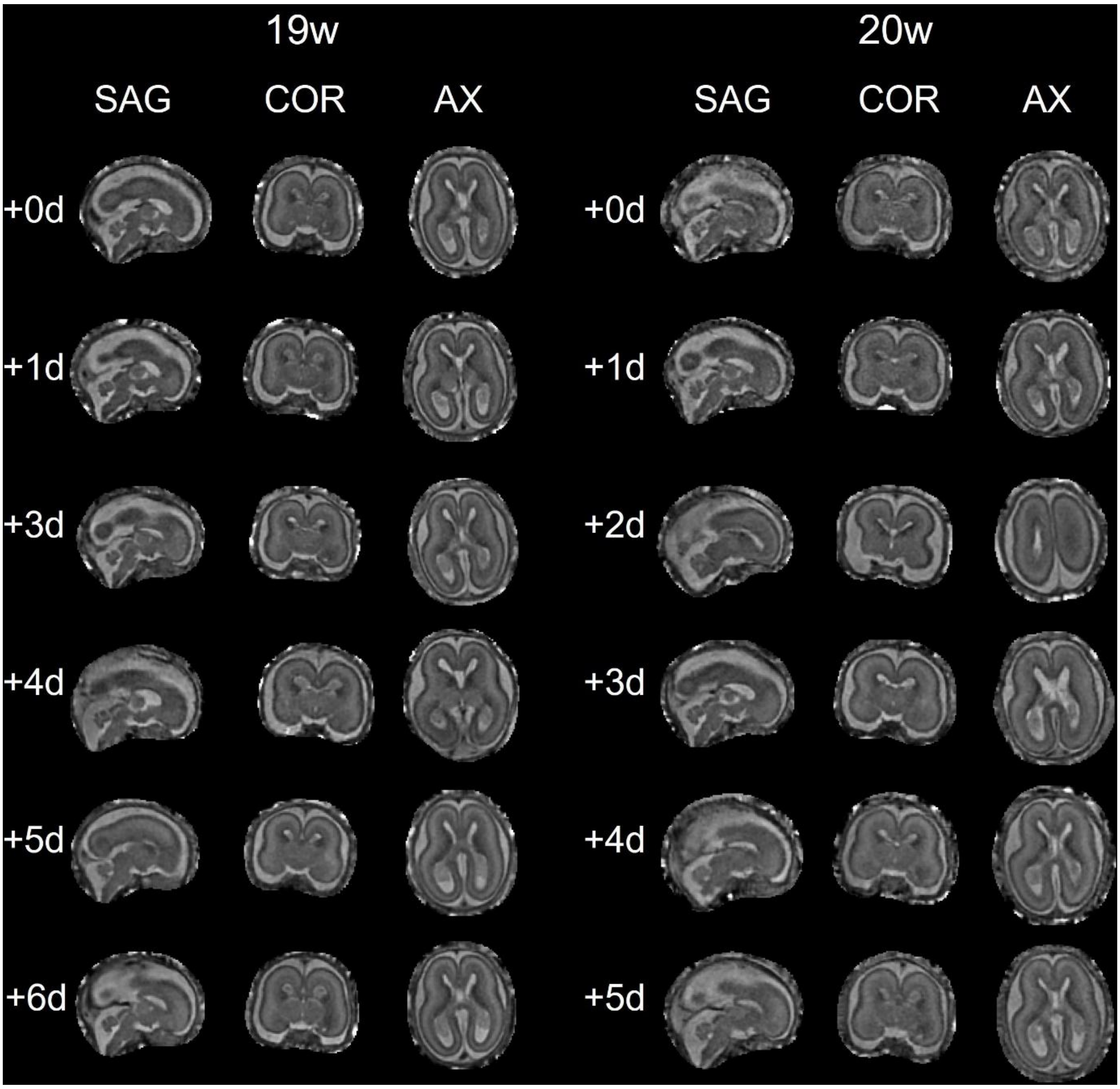

Figure S1: Image quality assessment of the fetuses included in atlas construction at 19 and 20 GWs. Each fetal T2w reconstruction is shown in the three orthogonal views (axial, coronal and sagittal). w = week, d = days

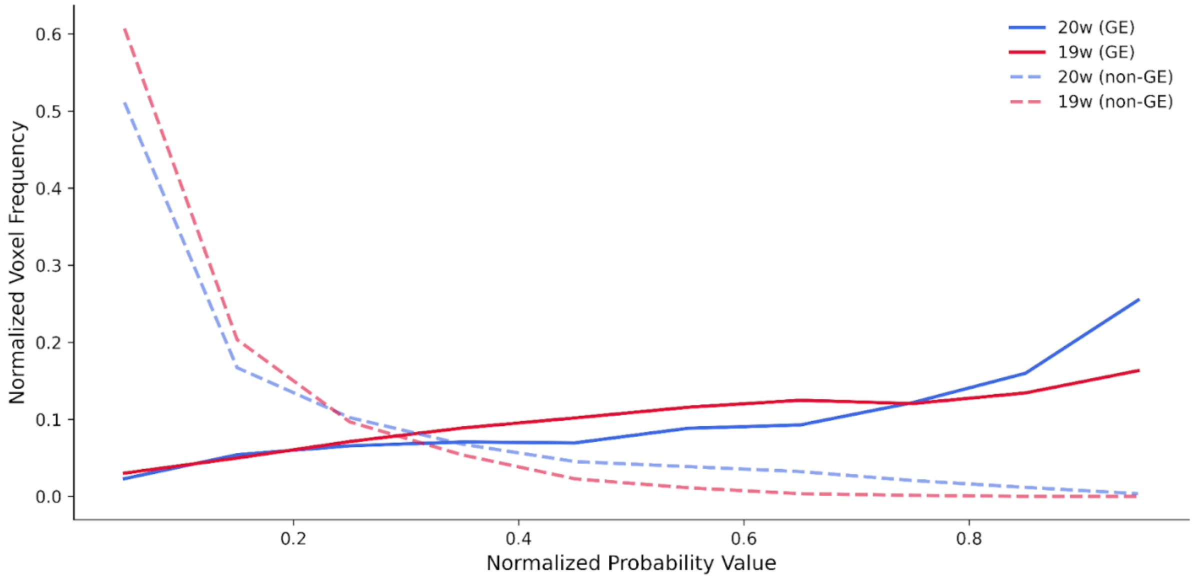

Figure S2: Normalized voxel intensity distributions of the GE probability maps at 19 and 20 gestational weeks. Solid lines represent voxels within the GE in the atlas space, while dashed lines correspond to voxels outside the GE mask in the atlas space. Probability values were normalized to the [0,1] range.

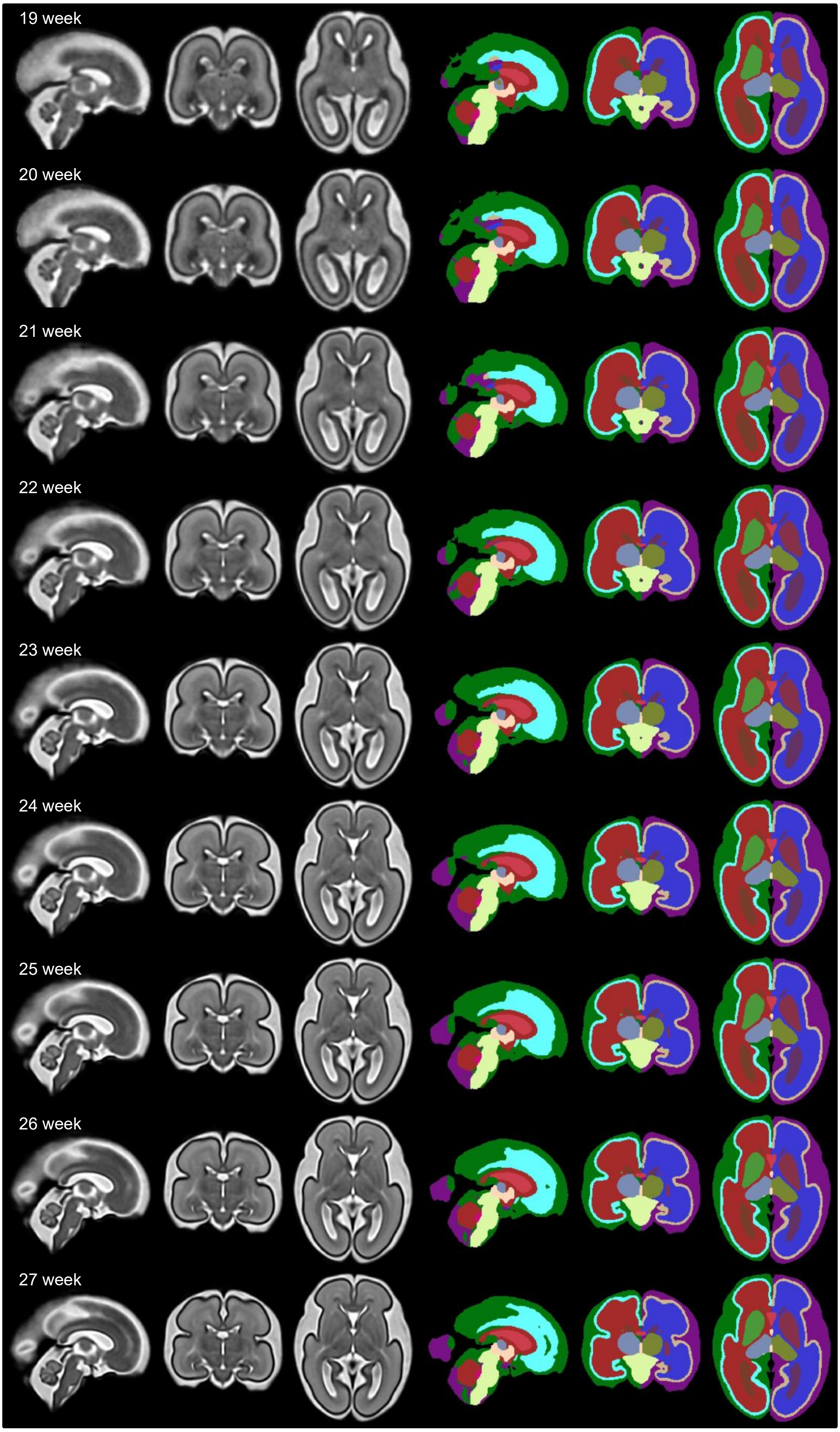
19 week
20 week
21 week
22 week
23 week
24 week
25 week
26 week
27 week

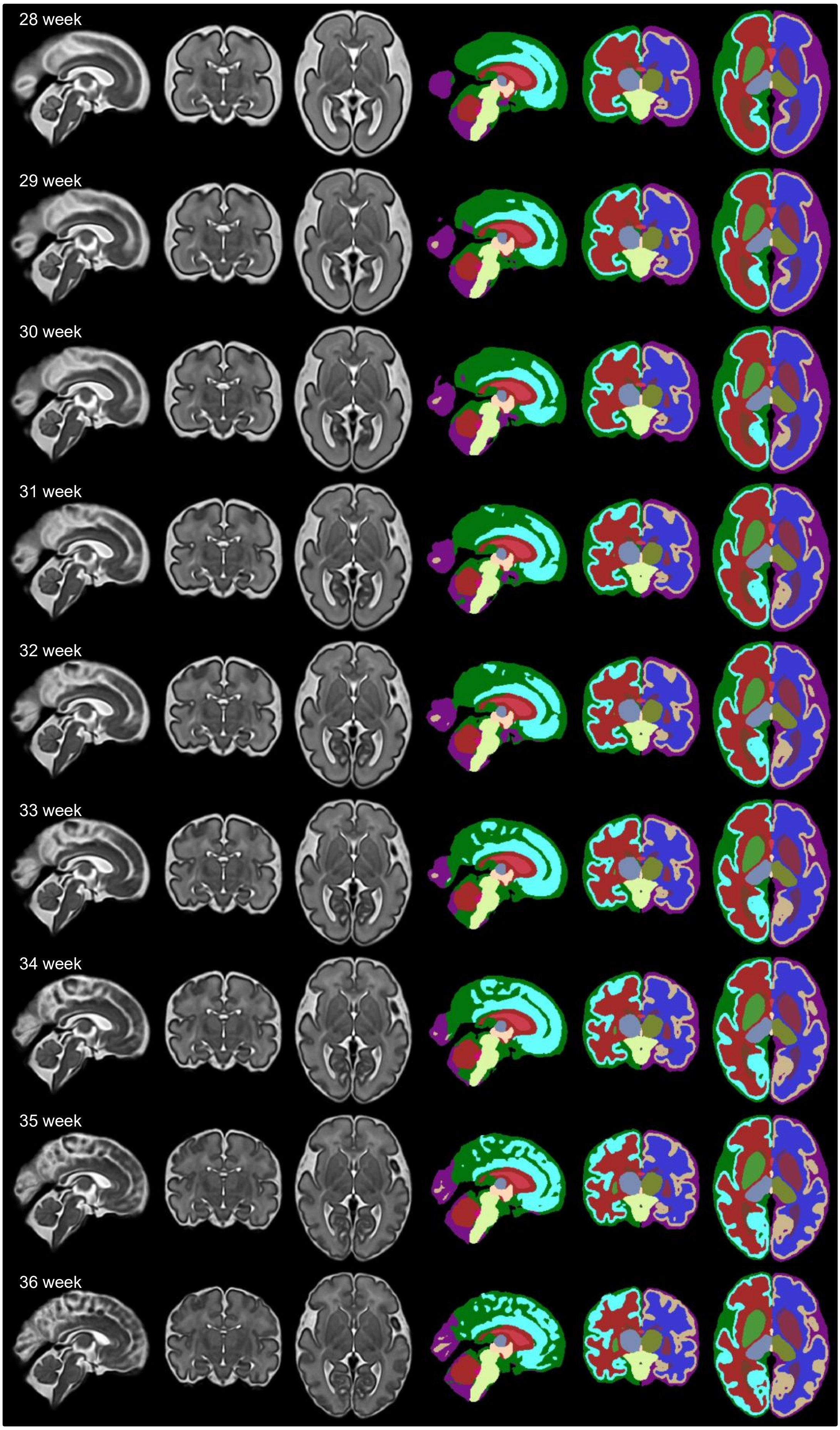
28 week
29 week
30 week
31 week
32 week
33 week
34 week
35 week
36 week

Figure S3: Atlas of fetal brains between 19-36 week of gestations. The structural images and the label maps are reported in the three orthogonal views (axial, coronal and sagittal) for each week.
